# Metastatic pheochromocytoma complicated with Langerhans cell histiocytosis: a case report

**DOI:** 10.3389/fendo.2025.1494783

**Published:** 2025-04-11

**Authors:** Dandan Dai, Jing Xie

**Affiliations:** Department of Pathology, Ruijin Hospital Affiliated to Shanghai Jiaotong University School of Medicine, Shanghai, China

**Keywords:** Langerhans cell histiocytosis, pheochromocytoma, metastasis, case report, EPAS1 gene

## Abstract

Pheochromocytoma is a neuroendocrine neoplasm that originates from chromaffin cells of the adrenal medulla. Langerhans cell histiocytosis (LCH) is a proliferative disease of histiocyte-like cells, often associated with activating mutations of the mitogen-activated protein kinase (MAPK) pathway. We present a case of a 49-year-old male with a history of pheochromocytoma, which metastasized to the inferior vena cava eight years after left adrenalectomy. At the same time, it was found that the pheochromocytoma in the metastasis was complicated with LCH, a combination that has not been previously reported. Genetic analysis was carried out by next-generation sequencing (NGS) technology. Somatic mutations of *BRAF* and *RAD54B* were detected in Langerhans cells and *EPAS1* in pheochromocytoma.

## 1 Introduction

Pheochromocytomas are rare tumors originating in the adrenal medulla (1) and usually secrete catecholamines leading to hypertension and myocardial degenerative effects (2). Metastatic Pheochromocytoma is most commonly reported in the local lymph nodes, bone, liver and lung (3). Since it is impossible to differentiate non-metastatic and metastatic Pheochromocytoma based upon clinical or even histopathological findings, all Pheochromocytoma are currently considered potentially metastatic tumours (WHO 2022 classification) (4). As a result, all patients with Pheochromocytoma require long and intensive follow up. Pheochromocytoma mostly results from pathogenic variants of predisposing genes, with a genetic contribution that now stands at around 70%. Germline variants account for approximately 40%, while the remaining 30% is attributable to somatic variants (5). Langerhans cell histiocytosis (LCH), the most common histiocytic disorder, encompasses conditions characterized by aberrant function and differentiation or proliferation of cells of the mononuclear phagocyte system (6). LCH is a histiocytic neoplasm characterized by a mass of CD1a + CD207+ histiocytes, exhibiting a diverse range of clinical manifestations from a self-healing rash or single bone destruction to multi-organ disease with potentially fatal consequences (7). In this article, we present a unique case of metastatic pheochromocytoma complicated with LCH. A 49-year-old male had undergone a left adrenalectomy in 2016 and pathology confirmed pheochromocytoma. In January 2024, the patient presented with intermittent low-grade fever, prompting a comprehensive examination that revealed a mass within the inferior vena cava. Elevated catecholamine levels were detected in both blood and urine. While high resolution CT showed multiple nodules in both lungs. The patient was considered to have multiple metastases of pheochromocytoma and a biopsy of the mass in the inferior vena cava was performed. The pathology confirmed metastatic pheochromocytoma complicated with LCH. Molecular pathology showed *EPAS1* mutation in pheochromocytoma. *BRAF* insertion mutation and *RAD54B* frameshift mutation was detected in in Langerhans cells. This report is, to our knowledge, the first case of pheochromocytoma coexisting with LCH, and also the primary report of detecting *RAD54B* mutation in LCH. It highlights the possibility of intravascular metastasis occurring simultaneously with LCH in patients with a previous history of pheochromocytoma even years after adrenalectomy and emphasizes the need to adopt a comprehensive next-generation sequencing (NGS) panel. According to the latest guidelines, it is mandatory to perform genetic analysis in all pheochromocytoma cases regardless of phenotype. Besides, We propose testing for *RAD54B* gene variants. A possible correlation between *RAD54B* pathogenic variants and LCH clinical course should be considered. For patients diagnosed with pheochromocytoma following surgical intervention, long-term blood pressure monitoring and regular follow-up are recommended to find recurrence or metastasis in time.

## 2 Clinical presentation

**Figure 1:**
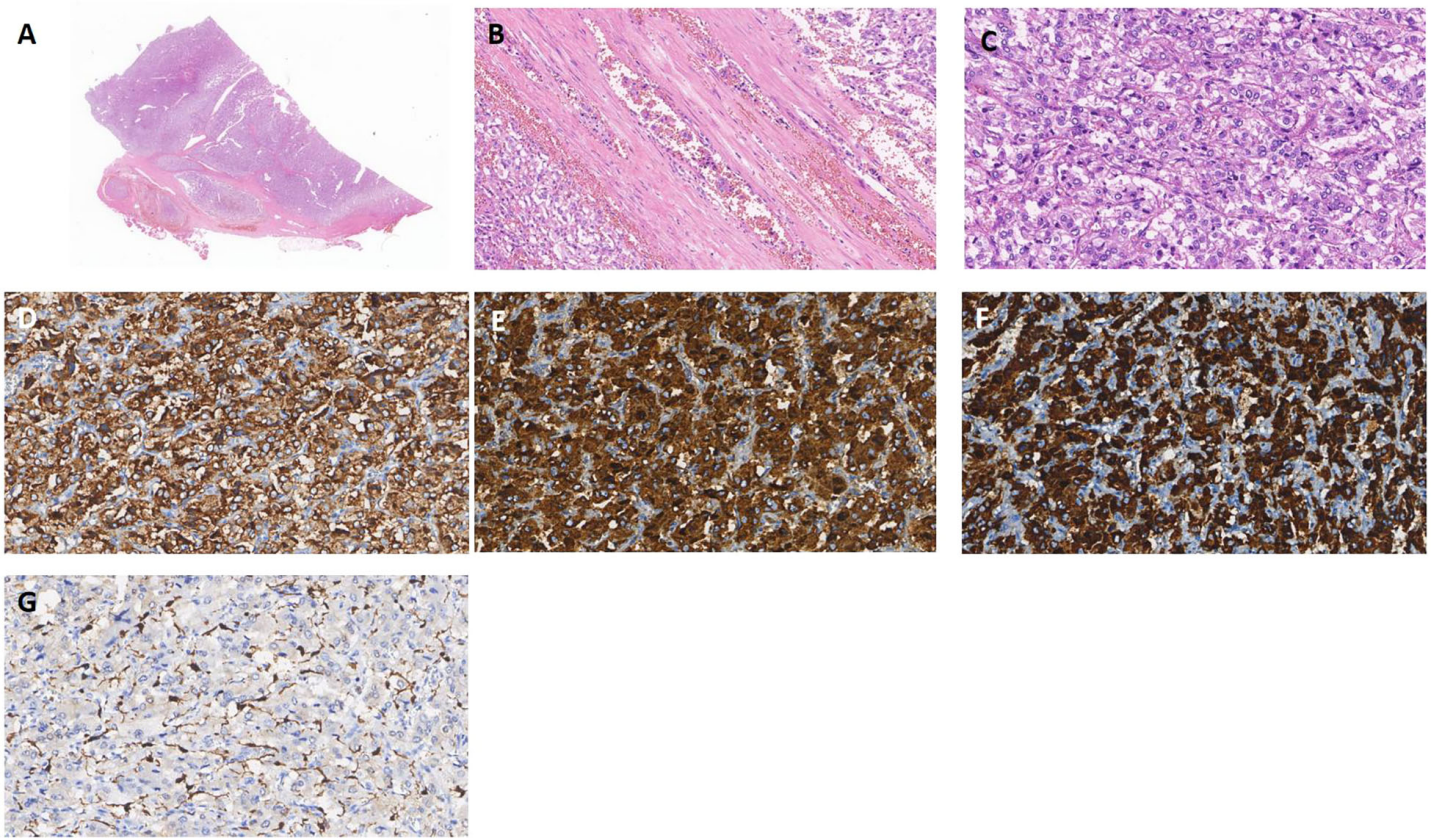
Pheochromocytoma **(A)** Pheochromocytoma with vascular invasion (x5 HE). **(B)** Intravascular tumor embolus (x100 HE). **(C)** Classic histoarchitecture with uniform cell nests(Zallballen)(x200 HE). **(D-F)** Syn, CgA and SDHB expression is positive, supporting the diagnosis of pheochromocytoma(x200). **(G)** Immunohistochemical stain for S100 showing classic distribution of sustentacular cells, mostly at the periphery of cell nests(x200).

A 49-year-old man without specific family history presented with nocturnal episodic headaches in 2016. The headache lasted about 2 hours, accompanied by sweating, no obvious palpitation or dizziness. The patient presented at the local hospital with elevated blood pressure (specific value unknown). Urinary measurements demonstrated elevated level of vanillylmandelic acid at 15.28 mg/24h (normal range, 2-6 mg/24 h), while aldosterone and serum calcium remained within normal limits. Abdominal computed tomography revealed a left adrenal nodule approximately 6.1x4.5cm in size. On the basis of these findings, pheochromocytoma was suspected. In the same year, the patient underwent a left adrenalectomy. The headache and sweating recovered soon after operation. The pathology examination revealed the presence of pheochromocytoma with vascular invasion and capsular invasion(**Figures 1A–C**). Tumour cells were strongly immunopositive for synaptophysin (**Figure 1D**), chromogranin A (**Figure 1E**), SDHB (**Figure 1F**). Sustentacular cells were immunopositive for S100 protein(**Figure 1G**). Moreover, immunostains for AE1/AE3, CD56, CD34, Her2, Inhibin and HMB45 were negative (data not shown). Pheochromocytoma of the Adrenal Gland Scaled Score (PASS) of 4 was assigned and the Ki67 proliferation index was 1%. According to the guidelines for genetic screening of Pheochromocytomas and paragangliomas (PPGLs), our patient was proposed for next-generation sequencing (NGS) targeting. *EPAS1* mutation was detected in pheochromocytoma. No germline mutations were found. The patient was treated with antihypertensive medication resulting in blood pressure control within the normal range. After discharge, the patient has not been regularly followed up.

In January 2024, the patient developed intermittent low-grade fever. He received treatment at the local hospital for 2 days without improvement (specific details unknown). Abdominal color Doppler ultrasound showed foreign bodies in the inferior vena cava, while high resolution CT showed multiple nodules in both lungs. 68Ga-DOTATATE PET/CT scan(0.59mCi) was performed showing a soft tissue density mass in the left renal vein-inferior vena cava with abnormally high metabolism and multiple solid nodules in both lungs with slightly high metabolism. Combined with the medical history, the patient was considered to have tumor recurrence invading the inferior vena cava, along with multiple metastases in both lungs. The patient’s low-grade fever resolved spontaneously after hospitalization and did not recur.

**Figure 2:**
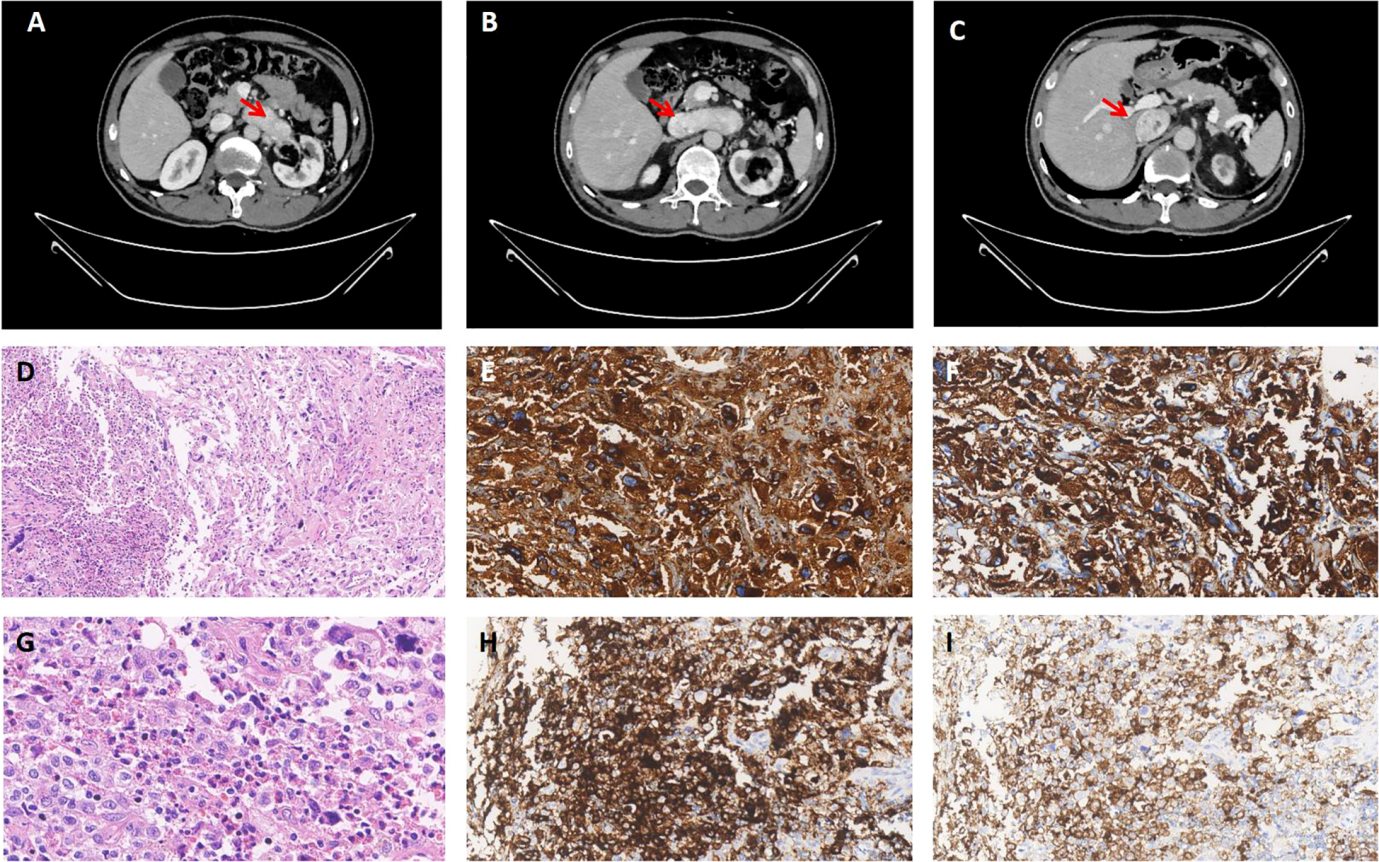
Computed tomographic angiography and pathological images. **(A-C)** The lumen of the left renal vein showed a soft tissue mass extending from its junction with the inferior vena cava, along the course of the inferior vena cava. The enhanced scan exhibited significant enhancement. Pheochromocytoma in metastasis. **(D)** The mononuclear cell area on the left side exhibits a significant presence of numerous eosinophils(x100 HE). **(E, F)** Pheochromocytoma expressing CgA and syn (x200). **(G)** The monocytic cells have reniform-to-oval nuclei and a central horizontal groove(x400 HE). **(H, I)** The mononuclear cells are positive for CD1a and Langerin (x200).

In March 2024, the patient came to the hospital for further examination. Laboratory tests revealed an elevated level of plasma normetanephrine at 5279.1 pg/ml (normal range, 19–121 pg/ml), while metanephrine remained within normal limits. Urinary measurements also revealed elevation levels of epinephrine at 22.57 ug/24h (normal range, <22ug/24h) and norepinephrine at 2290.15 ug/24h (normal range, 7-65ug/24h). Computed tomographic angiography of the abdominal aorta revealed the presence of a mass extending from the left renal vein to the inferior vena cava (**Figures 2A–C**). No obvious abnormality was found in the right adrenal gland. After two weeks of prophylactic treatment with alpha-blockers, a biopsy of the tumor in the inferior vena cava was conducted under local anesthesia. Pathology confirmed metastatic pheochromocytoma accompanied by LCH. Immunophenotyping of chromaffin cells was consistent with the primary lesion (**Figures 2E, F**). The pheochromocytoma area was accompanied by an adjacent region comprising of large monocytic cells with reniform-to-oval nuclei and a central horizontal groove, and plentiful of eosinophils (**Figures 2D, G**). The mononuclear cells revealed positivity for CD1a (**Figure 2H**), Langerin (**Figure 2I**) S100 and the Ki67 proliferation index was 10%. Molecular pathology identified the c.1457_1471del; p.N486_P490del mutation in the 12th exon of the *BRAF* gene, and the c.528dupT; p.V177Cfs*9 mutation in the 5th exon of the *RAD54B* gene. The patient underwent a 68Ga-DOTATATE PET/CT scan(0.59mCi). Multiple solid pulmonary nodules were found in both lungs, some of which had slightly higher metabolism. It may suggest the potential pulmonary metastasis from pheochromocytoma. The patient was recommended to undergo further diagnostic tests in order to confirm the diagnosis. However, the patient expressed no intention to pursue additional investigations. Considering the difficulty of the operation, the patient postponed the operation and took antihypertensive treatment including doxazosin mesylate extended release tablets and arotinolol hydrochloride tablets. The 24-hour ambulatory blood pressure showed that systolic blood pressure and diastolic blood pressure were normal during the day and night after drug use. The circadian rhythm of systolic blood pressure and diastolic blood pressure disappeared. The 24-hour systolic blood pressure and diastolic blood pressure increased (120/77mmHg). After discharge, the patient’s blood pressure was monitored and stabilized, and regular follow-up was requested. The clinical course is shown in **Table 1**.

**Table 1:** Clinical timeline.

Time	Diagnostic Findings	Treatment	Pathological Diagnosis
2016	- Elevated blood pressure (value unspecified)	Left adrenalectomy	Pheochromocytoma with intravascular tumor embolus and capsular invasion
	- Elevated urinary vanillylmandelic acid (VMA)		
	- Left adrenal nodule (6.1×4.5 cm) on abdominal CT		
January 2024	- Foreign bodies in inferior vena cava (abdominal ultrasound)	N/A	N/A
	- Multiple bilateral lung nodules (high-resolution CT)		
	- 68Ga-DOTATATE PET/CT: High-metabolism mass in left renal vein-IVC; lung nodules		
March 2024	- Elevated plasma normetanephrine and urinary epinephrine/norepinephrine	- Tumor biopsy	Metastatic pheochromocytoma with LCH (Langerhans cell histiocytosis)
	- IVC mass extending from left renal vein (CT angiography)	- Antihypertensive therapy	

## 3 Discussion

This case represents a rare complication of pheochromocytoma metastasizing to the inferior vena cava with concomitant LCH. The metastasis of pheochromocytoma to the inferior vena cava has not been reported. In this case *EPAS1*(*HIF2α*) mutation was detected. *EPAS1*has been identified as one of the susceptibility genes associated with Pheochromocytomas (8).

PPGLs are rare neuroendocrine tumors (NETs) derived from adrenomedullary chromaffin cells and from the autonomic paraganglia, respectively. Pheochromocytomas (PCCs) represent about 80–85% of chromaffin-cell neoplasms, whereas paragangliomas (PGLs) account for the remaining 15–20%. Based on transcriptional profile, PPGLs are classified into three clusters. The tree clusters are: 1) hypoxia/pseudohypoxia, 2) kinase signaling group, 3) Wnt signaling pathway (9). Cluster 1 includes PPGLs with variants in genes encoding the hypoxia-inducible factor (HIF) 2α, the Von Hippel–Lindau tumor suppressor (VHL), the prolyl hydroxylase domain (PHD), fumarate hydratase (FH), and succinate dehydrogenase subunits (SDHx) (10). All these mutations promote HIFa stabilization and accumulation resulting in increased angiogenesis via changes in vascular endothelial growth factor-1 and -2 receptors (VEGFR1/2) and platelet-derived growth factor-b receptor (PDGFR) transcription (11). Cluster 2 consists of germline or somatic mutations in RET, NF1, TMEM127, MAX, HRAS and KIF1Bβ which associated with PI3 kinase pathways the “PI3K/AKT/mTOR and MAPK/ERK”. Cluster 2 PPGLs are mostly benign exhibiting a mature catecholamine phenotype (12). Cluster 3 PPGLs are due to somatic mutations of the CSDE1 gene or somatic gene fusions of the MAML3 gene (13). Cluster 3 tumors have a more aggressive behavior (5).

LCH is caused by clonal expansion of myeloid precursors that differentiate into CD1a+/CD207+ cells in lesions that leads to a spectrum of organ involvement and dysfunction (14). LCH is often characterized by activating mutations of the mitogen-activated protein kinase (MAPK) pathway with *BRAFV600E* being the most recurrent mutation. The remaining cases of LCH that do not bear the *BRAFV600E* mutation are often characterized by other mutations in the *BRAF* gene (15). *BRAF* mutations have been reported in >50% of patients with LCH (16). BRAF is a core component of the MAPK/ERK1/2 signaling cascade and involves the sequential phosphorylation and activation of RAS-RAF-MEK-ERK (17). In this case, molecular pathology identified the c.1457_1471del; p.N486_P490del mutation in the 12th exon of the *BRAF* gene, which is atypical.

Notably, for the first time, a somatic mutation in the oncogene *RAD54B* was identified in Langerhans cells. RAD54B belongs to the SNF2/SWI2 superfamily, involving in cell cycle regulation after DNA damage and participating in homologous recombinational repair, which ensures the precise repair of the most deleterious DNA lesions, double-stranded breaks (18). *RAD54B* displays oncogene-like characteristics and is amplified or overexpressed in a diverse array of cancer types, including colorectal, lung, prostate and breast (19). It has not been proven yet that *RAD54B* mutation is associated with LCH.

Tumorigenesis is a multiphase process dependent on several modifications at cellular and tissue levels, leading to sustain proliferative signalling, evasion from growth suppressors and from cell death, replicative immortality, and induction of angiogenesis, invasion, and metastasis (20). Beyond genetic alterations, the interplay among cancer cells and tumour microenvironment (TME) components has a central role in tumour initiation and progression (21). Angiogenesis, the development of new blood vessels from established vasculature, provides growth and hematogenous dissemination of the cancer cells. In this case EPAS1 mutation was detected in pheochromocytoma. EPAS1 mutation promotes HIFa stabilization and accumulation resulting in increased angiogenesis, which might enhance the likelihood of the formation and metastasis of other tumors, resulting in the coexistence of the two types of tumors.

More conclusive evidence is still required to ascertain whether there is a definitive correlation between pheochromocytoma and LCH concerning their pathogenesis. Currently, it remains uncertain whether this case represents a simple collision tumor or if it is an instance of LCH triggered by a pheochromocytoma. Due to the presence of multiple nodules in lungs, we were more inclined to suspect that the patient may have systemic LCH and recommended further examination. However, the patient did not cooperate. We recommend regular monitoring of blood pressure and periodic reviews for the patient.

Presently, clinical and hystopatological scoring systems have been studied and validated to assist in predicting the risk of disease recurrence. Parasiliti-Caprino et al. (22) conducted a retrospective multicenter study on 177 PCC patients who underwent radical surgery and proposed a multivariable continuous model for post-surgical PCC recurrence prediction. The model was named the SGAP-model (size, genetic, age, and PASS). It was created on an 8-point scale, by assigning 1 point for tumor size > 50 mm, 3 points for positive genetic testing, 1 point for age ≤ 35 years, and 3 points for PASS ≥ 3. Patients with a SGAP-score of 0-2 showed a virtually absent risk of recurrence; patients with a SGAP-score of 3-4 showed an intermediate risk profile; patients with a SGAP-score of 5-8 showed a markedly elevated risk of recurrence that exceeded 60% after 10 years. An accurate estimation of recurrence risk would be of fundamental importance in clinical practice, as it may allow clinicians to suggest a higher-intensity monitoring when the estimated recurrence risk is high.

## 4 Conclusion

In conclusion, the current case reminds us that pheochromocytoma can metastasize to the inferior vena cava. Due to its rare occurrence and non-specific clinical manifestations, imaging may still be the most valuable method for discovering metastasis. Pheochromocytoma accompanied by LCH has not been reported yet. This rare complication may indicate a potential relationship in the pathogenesis between the two. Whether *RAD54B* gene mutation is a pathogenic mutation of LCH needs to be further explored. Importantly, no pheochromocytoma can be considered fully benign and all patients should be followed for life for recurrence, new primary pheochromocytoma, and metastatic disease.

## Data Availability

The original contributions presented in the study are included in the article/supplementary material. Further inquiries can be directed to the corresponding author.
